# Dietary Quality in Vegetarian and Omnivorous Female Students in Germany: A Retrospective Study

**DOI:** 10.3390/ijerph18041888

**Published:** 2021-02-16

**Authors:** Julia Blaurock, Birgit Kaiser, Tamara Stelzl, Michelle Weech, Rosalind Fallaize, Rodrigo Zenun Franco, Faustina Hwang, Julie Lovegrove, Paul M. Finglas, Kurt Gedrich

**Affiliations:** 1 ZIEL-Institute for Food & Health, Technical University of Munich, 85354 Freising, Germany; julia.blaurock@tum.de (J.B.); birgit.kaiser@tum.de (B.K.); 2 Analytical Food Chemistry, Technical University of Munich, 85354 Freising, Germany; tamara.stelzl@tum.de; 3 Hugh Sinclair Unit of Human Nutrition, Department of Food and Nutritional Sciences, University of Reading, Reading RG6 6DZ, UK; m.weech@reading.ac.uk (M.W.); r.fallaize@reading.ac.uk (R.F.); j.a.lovegrove@reading.ac.uk (J.L.); 4 Globalyze, Virgílio Malta 17-76, 17014-440 Bauru, Brazil; rodrigo@globalyze.work; 5 Biomedical Engineering, School of Biological Sciences, University of Reading, Reading RG6 6DH, UK; f.hwang@reading.ac.uk; 6 Quadram Bioscience Institute, Norwich NR4 7UQ, UK; paul.finglas@quadram.ac.uk

**Keywords:** vegetarian, omnivores, nutrient intake, food frequency questionnaire, healthy eating index, web application, personalized dietary advices

## Abstract

Vegetarian diets have gained in popularity, especially among highly educated women, and are considered beneficial to health. Comparative studies assessing the diet of vegetarians against omnivores are rather limited and often provide ambivalent results. Therefore, this study examined the nutrient intake and nutritional quality of vegetarian and omnivorous diets in a group of 61 female students in Germany. Habitual dietary intake was evaluated using a validated graphical online food frequency questionnaire (FFQ). Differences in nutrient intakes were analyzed by Mann–Whitney-U-Tests. Odds Ratios (OR) were calculated for vegetarians exceeding dietary reference values (DRV) compared to omnivores. The overall nutritional quality was assessed using the Healthy-Eating-Index-2015 (HEI-2015). In omnivores, intakes of total energy from saturated fatty acids (SFA), monounsaturated fatty acids (MUFA), long-chain omega-3 polyunsaturated fatty acids (LC-n3-PUFA), cholesterol, sucrose, lactose, retinol, and cobalamin were significantly higher than in vegetarians. Significantly lower intakes were observed for fiber, magnesium, and beta-carotene. Significant OR were detected for total fat (OR = 0.29), SFA (OR = 0.04), beta-carotene (OR = 4.55), and cobalamin (OR = 0.32). HEI-2015 scores were higher for vegetarians than for omnivores (79 points versus 74 points) and significant differences were recorded for the HEI-2015 components dairy, seafood & plant proteins, fatty acids, added sugars, and saturated fatty acids.

## 1. Introduction

A balanced vegetarian diet is often associated with a higher intake of vegetables, fruits, seeds, and nuts. Choosing a vegetarian diet can be associated with ethical, ecological, religious, or health motives [1]. Moreover, it has been shown that the probability of being vegetarian is positively associated with educational level and negatively associated with disposable per capita income [2]. There is evidence that a vegetarian diet supports positive health outcomes and can reduce the risk of several chronic diseases like ischemic heart disease or diabetes [3,4,5]. It was shown that vegetarians tend to better meet the dietary reference values (DRV) of the German Nutrition Society (DGE) than omnivores [6]. Due to a higher intake of plant-based foods and the absence of meat and fish, vegetarians were reported to have a lower energy intake and a more beneficial fat profile with lower intakes of cholesterol, total, and saturated fat and higher intakes in polyunsaturated fats [7]. Furthermore, the intake of vitamins associated with a plant-based diet, such as vitamin A, vitamin B1, vitamin C, vitamin E, and minerals, such as magnesium and potassium, as well as dietary fibers and secondary plant substances is often higher among vegetarians than in omnivores [3,8,9].

However, a restrictive vegetarian diet may also pose health risks due to reduced intakes of certain essential nutrients, such as iron, zinc, omega-3 polyunsaturated fatty acids, riboflavin, cobalamin and calcium [1,3,9]. Though, study results on the supply of these nutrients are ambiguous and influenced by the foods excluded in the respective diets [1,7,10,11]. Since nutrients are consumed as a combination of foods rather than in isolation, an assessment of single nutrients may not be sufficient. Considering the interactions of nutrients and the complexity of diets, it may be useful to additionally investigate nutritional patterns by means of a diet quality score [7].

A vegetarian diet is particularly popular among young people in Germany. In 2011, approximately 4% of the German population aged 18–79 were vegetarians [12]. They were particularly prevalent in the age group of 18–29 years, of which 9% of women and 5% of men followed a vegetarian diet. Nutritional information on vegetarian female students is scarce. In a study of dietary patterns among students in Germany, it was shown that meat consumption of male students was on average twice as high as meat consumption of female students [13]. Due to their young age and high educational level, female students form an interesting group to compare vegetarian and omnivorous diets.

To our knowledge, there are currently no studies comparing the diet between female vegetarian and non-vegetarian students in Germany. Therefore, the aim of the present analysis was to address the research questions whether female vegetarian students (1) eat healthier and (2) meet the DRV better than omnivorous students. To answer these questions, female students living in Germany were split into vegetarians including those with occasional fish consumption (*n* = 3) and omnivores based on their self-declaration within a pre-study questionnaire. Nutrient intakes and dietary quality was assessed using a novel web-based eNutri2019 food frequency questionnaire (FFQ). The latter was developed as part of the EIT-Food Quisper project. Quisper (Quality Information Services and Dietary Advice for Personalized Nutrition in Europe) is a digital platform that brings together scientifically robust personalized nutrition services and data [14,15]. The eNutri2019 FFQ and its web application were developed for assessing individuals’ dietary habits as a potential basis to deliver personalized dietary advices via the Quisper Platform (https://quisper.eu/, accessed on 15 February 2021). The paper contributes to the scarce literature on nutrient intakes and nutritional quality of vegetarian female students who represent a large proportion of vegetarians.

## 2. Materials and Methods

### 2.1. Study Design

As part of the eNutri2019-study, a nationwide online study of 4 weeks, the food intake of 61 female university students living in Germany was examined more closely. A total of 31 vegetarians and 30 omnivores were included in the study. Ethical approval for the study was granted by the Research Ethics Committees of the Technical University Munich (approval no. 328/19S). Students were recruited via social media, and applicants were required to sign written consent forms prior to study participation. Specific medical conditions (e.g., diabetes, pregnancy) as well as specific diets (e.g., vegan, sport-specific diets) and an age below 18 years were defined as exclusion criteria. Furthermore, two study participants reporting an energy intake of more than 4500 kcal per day were considered as over-reporters and eliminated from the dataset. After elimination of the outliers, energy intakes ranged from 706 kcal to 3646 kcal per day.

### 2.2. Dietary Assessment

Habitual dietary intakes of participants were assessed using a novel self-administered, online FFQ as part of the eNutri2019-DE web application. A detailed functioning of the web application is described elsewhere [16]. The FFQ was based on the food list and portion size images of a previously validated FFQ from the Food4Me study, which was found to be reproducible across seven European countries, including Germany [17,18,19]. Based on the FFQ from the Food4Me study, the eNutri FFQ was further adapted with the inclusion of updated food items, portion sizes, portion size images, and the option to choose from seven different portion sizes [20]. To capture food intake frequencies, study participants could choose between the following options: <1 time/month (mo), 1–3 times/mo, 1 time/week (wk), 2–4 times/wk, 5–6 times/wk, 1 time/day (d), 2–3 times/d, 5–6 times/d, or ≥7 times/d. The eNutri2019-FFQ covers a total of 159 food items representative of the German diet, incorporating popular country-specific foods such as traditional German breads, sausages, fermented cabbage, side dishes and desserts. Sixteen food items focused on meat consumption and therefore were skipped for the vegetarians. Calculations of nutrient intakes in the web application were based on consumed quantities and matched with the German Nutrient Database (Bundeslebensmittelschlüssel, *BLS*) V.3.0.1 underlying the questionnaire [21].

### 2.3. Overall Dietary Quality

The overall dietary quality of vegetarian and omnivorous subjects was evaluated based on the Healthy Eating Index-2015 (HEI-2015). The HEI-2015 was calculated in accordance with the guidelines of the U.S. Department of Health and Human—National Cancer Institute (NIH) and comprised 13 components including total fruits, whole fruits, total vegetables, greens and beans, whole grains, dairy, total protein foods, seafood and plant proteins, fatty acids, refined grains, sodium, and added sugar [22]. Based on nutrition information provided by the study participants in the FFQ, intakes of individual foods and nutrients were quantified with reference to the respective HEI-2015 components and the German Nutrient Database [21]. Depending on dietary intake, a maximum of 5 or 10 points was awarded to individual HEI-components and summed up to a total score of maximum 100 points. In orientation to the Dietary Guidelines for Americans (DGAs) [23], high HEI-2015 scores (>81 points) indicate “good” nutritional quality, while low scores (≤50) imply “poor” diets [24]. HEI-2015 values of 51–80 points are indicative of a diet that “needs improvement”, with low compliance to dietary recommendations.

### 2.4. Physical Activity

The General Practice Physical Activity Questionnaire (GPPAQ) was used to assess the subjects’ physical activity as part of the eNutri2019 web application. It is a validated screening tool for measuring the physical activity levels of adults (16–74 years). The results of the tool are categorized into a four-level Physical Activity Index (PAI): Active, Moderately Active, Moderately Inactive, and Inactive [25].

### 2.5. Anthropometric and Socio-Economic Measurements

Participants were provided with instructions to accurately measure their height and weight themselves and these were self-reported on the eNutri2019 web application, from which their BMI was automatically calculated. Furthermore, socio-economic data was collected via a screening questionnaire in the web application.

### 2.6. Statistical Analysis

Non-parametric Mann–Whitney-U-Tests were used to compare the estimated nutrient intakes of vegetarian and omnivorous students. Significance was tested two-tailed, applying a 5% level of significance. Odds ratios (OR) were calculated to compare the compliance of vegetarians and omnivores with the DRV. The OR indicate the chance of vegetarians exceeding the DRV compared to omnivores. OR less than 1 indicate that the chance for vegetarians exceeding the DRV is smaller as compared to omnivores, while OR greater than 1 indicate that vegetarians are more likely to exceed the DRV as compared to omnivores. In order to calculate the OR, a two-by-two table was used [26]. Omnivores served as reference group. To detect large deviations from the DRV, proportions of participants with a nutrient intake of more than 100% and less than 67% of the DRV were determined.

## 3. Results

### 3.1. Study Participants

The sample comprised 61 female university students living in Germany, aged 18–30 years (mean = 22.5, SD = 4.07). Thirty-one women self-reported vegetarianism and were assigned to the ‘vegetarian’ group, and 30 women were assigned to the ‘omnivorous’ group. Mean age between the vegetarian group (25 y., SD = 17.7) and the omnivorous group (20 y., SD = 14.1) did not differ significantly (*p* = 0.40). Table 1 displays the main characteristics of the study participants. The majority of the students were of normal weight (BMI: ≥18.5 to <25 kg/m²), with the omnivorous group having a slightly higher mean BMI score (BMI: 22.5 kg/m²) than vegetarians (BMI: 21.9 kg/m²). However, the BMI did not differ significantly between the two groups (*p* = 0.37). Applying the PAI, the majority of both groups was categorized as ‘active’, that is, 65% of the vegetarians and 63% of the omnivores, resulting in a non-significant difference between the two groups (*p* = 0.95). Furthermore, both groups had no significant difference in income (*p* = 0.87): The majority of students had an income below 10.000€ per year (vegetarians 42%, omnivores 27%). Seventy-four of the vegetarians and 63% of the omnivores reported consuming dietary supplements such as omega-3 polyunsaturated fatty acids (vegetarians 3.2%, omnivores 6.7%), vitamin C (vegetarians 9.7%, omnivores 10.0%), vitamin D (vegetarians 25.8%, omnivores 13.3%), calcium (vegetarians 0.0%, omnivores 6.7%) and iron (vegetarians 25.8%, omnivores 20%).

### 3.2. Nutrient Intake

Energy and nutrient intakes from food and dietary supplements with respect to type of diet are shown in Table 2 along with the respective German DRV for nutrient intake. There were no significant differences in total energy intake between vegetarian and omnivorous students. Though, vegetarian students had a significantly lower total energy intake from fats (%TE) compared to omnivorous students. In particular, intakes were significantly lower for saturated fatty acids (SFA), the long-chain fatty acids palmitic acid (C16:0) and stearic acid (C18:0), monounsaturated fatty acids (MUFA, %TE), long-chain omega-3 polyunsaturated fatty acids (LC-n3-PUFA), and cholesterol. The same holds true for the intake of sucrose and lactose, as well as the (pro-)vitamins retinol and cobalamin. Conversely, the intakes of dietary fiber, magnesium, and beta-carotene were significantly higher among vegetarians.

### 3.3. Odds for Meeting DRV

**Figure 1:**
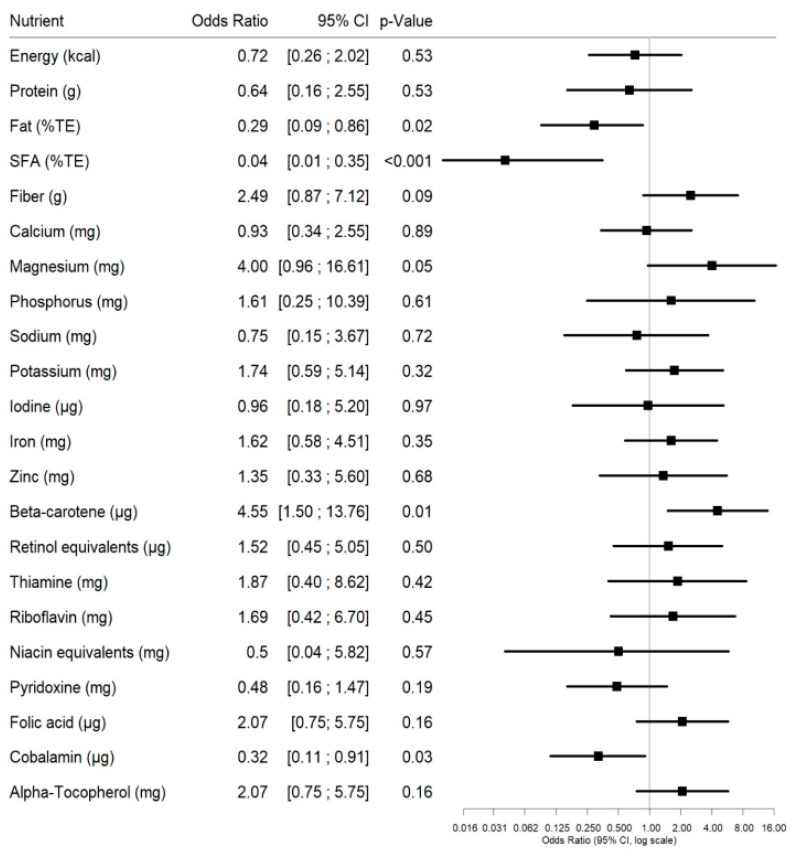
Forest plots showing odds ratios, 95% confidence intervals, and *p*-values (resulting from chi-squared tests) for selected nutrients. OR indicate the chance of vegetarians exceeding the dietary reference values (DRV) compared to omnivores.

Figure 1 shows the OR with their respective 95% confidence intervals for assessed nutrients. Compared to omnivores, vegetarians had a significantly lower chance of exceeding the DRV for total energy from fat (OR = 0.29, CI = 0.09;0.86), saturated fatty acids (SFA: OR = 0.04, CI = 0.01;0.35), and cobalamin (OR = 0.32, CI = 0.11;0.91). An OR of 0.29 means for example that the chance that vegetarians will exceed the DRV for fat is only 0.29 times the probability that relates to omnivores. For beta-carotene, the chance for exceeding the DRV was significantly higher for vegetarians compared to omnivores (OR = 4.55, CI = 1.50;13.76). For fiber and magnesium, OR were also very high (OR = 2.49, CI = 0.87;7.12, and OR = 4.00, CI = 0.96;16.61), though without reaching statistical significance.

### 3.4. Comparison of Nutrient Intakes with DRV

**Figure 2:**
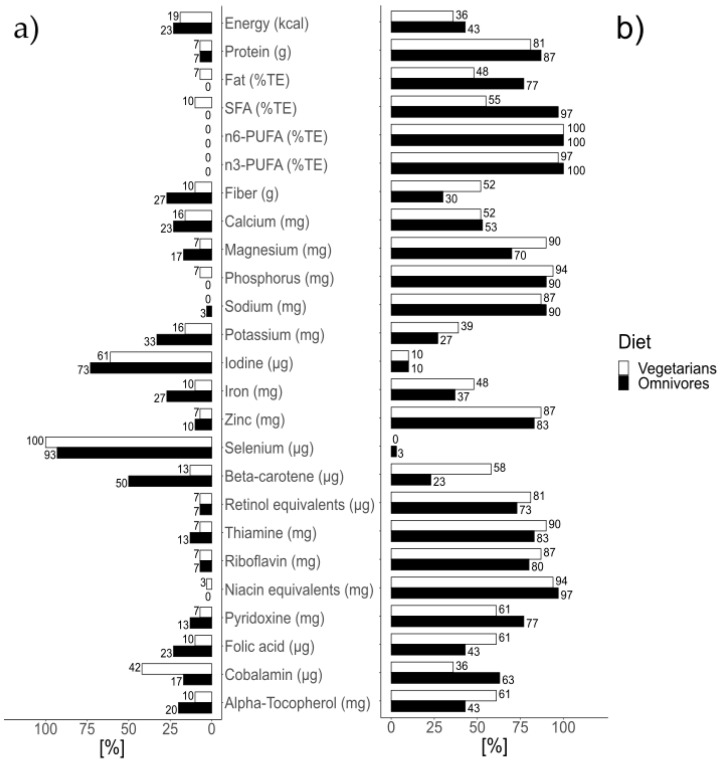
Proportions of participants (**a**) with an intake below 67% of the dietary reference values (DRV), (**b**) with an intake exceeding the DRV.

Proportions of participants who reached more than 100% or less than 67% of the DRV were notably extreme for n6-PUFA, n3-PUFA, phosphorus, sodium, selenium, and niacin equivalents. For n6-PUFA and n3-PUFA, none of the vegetarians and omnivores exhibited an intake less than 67% of the DRV and nearly all of them exceeded the DRV (Figure 2). For phosphorus, 7% of the vegetarians and none of the omnivores had an intake less than 67% of the DRV, while 94% of the vegetarians and 90% of the omnivores surpassed the DRV. For sodium, none of the vegetarians and 3% of the omnivores had an intake less than 67% of the DRV, and 87% of the vegetarians and 90% of the omnivores exceeded the DRV. Regarding selenium, all of the vegetarians and 93% of the omnivores had an intake less than 67% of the DRV, whereas none of the vegetarians and 3% of the omnivores exceeded the DRV. For niacin equivalents, 3% of the vegetarians and none of the omnivores had an intake less than 67% of the DRV, and 94% of the vegetarians and 97% of the omnivores surpassed the DRV.

### 3.5. HEI-2015

Female vegetarian students had an insignificantly higher average HEI-2015 total score of 79 points (out of a maximum of 100 points) than their omnivorous counterparts with 74 points (Table 3). Significant differences in nutritional behavior between both groups were specifically observed for the adequacy components ‘dairy’, ‘seafood & plant proteins’, ‘fatty acids’, and the moderation components ‘added sugars’ and ‘SFA’. As compared to omnivores, vegetarians consumed on median about 50% less dairy products, 16% less added sugars, 54% less SFA, 2% more seafood & plant proteins, and 30% more unsaturated fats. Looking at the maximum scores to be achieved, potential for improvement was particularly evident in vegetarians for the intakes of ‘dairy’, ‘protein foods’, ‘unsaturated fats’ (fatty acids), ‘refined grains’, ‘sodium’, and ‘SFA’. Potential for improvement in omnivores can be found in the components ‘total protein foods’, ‘seafood & plant proteins’, ‘fatty acids’, ‘refined grains’, ‘sodium’, ‘added sugars’, and ‘saturated fats’.

## 4. Discussion

### 4.1. HEI-2015

A vegetarian diet is associated with numerous positive health outcomes [27,28,29,30] and higher nutritional quality [31]. This study has shown that the dietary quality of German vegetarian students exceeded that of omnivores, which is in line with earlier results from observational studies. A cross-sectional survey on food consumption in Flemish adults in 2013, for example, reported an average HEI-2010 of 59 for vegetarians and 54 for omnivores [7]. A follow-up study with a matched-subjects design using a 3-day food diary, also found a significantly higher HEI-2010 total score for vegetarians (HEI: 54) compared to omnivores (HEI: 46) [32]. The National Health and Nutrition Examination Survey (NHANES; 2007–2012), assessing the nutritional status of the U.S. population, found average HEI-2010 scores of 73 for vegetarians and of 56 for meat eaters of different ethnicities [33]. It is worth noting that the HEI-2010 comprises 12 components and represents the predecessor version of the HEI-2015 launched in 2015. Compared to the HEI-2010, the HEI-2015 additionally considers the components ‘added sugars‘ and ’saturated fats‘, while disregarding the previous component of ‘empty calories’. Accordingly, the results of HEI-2010 are not fully comparable to those of the HEI-2015.

Taking the European follow-up study from Clarys et al. for instance [32], considerably lower HEI-2010 scores were observed for vegetarians and omnivores as compared to the HEI-2015 in the present study. When comparing the average scores of corresponding components between the HEI-2010 and HEI-2015, the German cohort consistently scored higher except for the two components ‘total protein foods‘ and ‘sodium‘ and therefore translates into a higher overall HEI-2015 score. As compared to the HEI-2010, the HEI-2015 has further been improved by inclusion of a legumes algorithm, designed to capture specific dietary habits such as plant-based diets more accurately [34]. Thus, the results of the HEI-2010 may deviate considerably from the HEI-2015. At the time of publication of this paper, however, there were no scientific studies available, investigating the dietary quality of vegetarians by applying the HEI-2015.

### 4.2. Macronutrients

As part of the study, the dietary intake of macro- and micronutrients was analyzed among the female student cohort. There is evidence from research that the energy intake and the BMI of vegetarians are significantly lower compared to omnivores [35,36,37]. Even though the current study did not reveal any differences in the average BMI between both dietary groups, discrepancies in macronutrient intakes were observed. Overall, the average energy (in kcal), protein (in g), and carbohydrate (in g) intakes of vegetarians were negligibly lower than for omnivores. However, vegetarians consumed significantly less energy from fat, less SFA, and lower amounts of cholesterol compared to non-vegetarians, which is consistent with previous results from the *European Prospective Investigation into Cancer and Nutrition-Oxford study* (*EPIC-Oxford*; 1993–1999), which compared the dietary intake of 4164 British female vegetarians and 11.238 meat eaters [38]. Looking at the different types of fats, significant differences were detected in the intake of the SFA palmitic acid (C16:0) and stearic acid (C18:0), which is coherent as these constitute the two major fatty acids in red meat [39]. Considerable differences were also noticed for the intake of LC-n3-PUFA. Krajčovičová-Kudláčková et al. (1997) compared plasma levels of total PUFAs in younger vegetarians with omnivores, while semi-vegetarians with occasional consumption of fish or meat had significantly increased levels of the fatty acid eicospentaenoic acid (EPA; C20:5) and slightly elevated levels of docosahexaenoic acid (DHA; C22:6), while no differences were found for arachidonic acid (ARA; C20:3) and dihomo-gamma-linolenic acid (DGLA; C20:4) [40]. Even though this study considered LC-n3-PUFA as a whole without differentiating between individual fatty acids, their intakes in vegetarians were significantly lower compared to omnivores. EPA and DHA are known to be mainly derived from the consumption of oily fish and to a lesser extent from meat and dairy products [41]. Considering that 58% of the vegetarians stated in their FFQ that they had not eaten any fish at all, and taking into account that the portion sizes of those who ate fish were on average 44% smaller than in omnivores, might explain the lower LC-n3-PUFA intake of vegetarians. In terms of carbohydrates, the average intakes of both vegetarians and omnivores did not reach the German DRV of 50% of total energy. Compared to omnivores, vegetarians consumed significantly less lactose and sucrose, while their fructose intake was slightly higher. The scientific information on carbohydrate intakes by vegetarians is contradictory. A study by Bradbury et al. (2017) showed that vegetarians consumed less food containing high levels of free sugar and eat more foods high in fructose (e.g., fruits, vegetables) [42]. Slattery et al. (1991) detected lower intakes of sucrose in vegetarians, while the *National Health and Nutrition Examination Survey* (*NHANES*; 1999-2004) found higher intakes of added sugar [43,44]. In the current study, vegetarians consumed significantly less sugar-rich foods (e.g., soft drinks, fruit juices, sweet pastries) and dairy products compared to omnivores, thus explaining the lower sucrose and lactose intakes. The increased consumption of fruit and vegetables by vegetarians is probably related to the marginally higher fructose intake. 

The median dietary fiber consumption was almost 30% higher in vegetarians than in omnivores, mainly due to the increased consumption of whole meal cereals, fruits, and vegetables, thus exceeding the respective DRV of >30 g/d. This is consistent with previous studies, also demonstrating a significantly higher fiber intake by approximately 15–30% among European female vegetarians [37,45].

### 4.3. Micronutrients

As for micronutrients, a significant difference in mean magnesium intake was observed between vegetarians and omnivores, both of which were well above the respective DRV of 300 mg/d. Evidence suggests that diets rich in vegetables and unrefined grains are higher in magnesium than diets including meat and dairy products [46,47]. It has also been shown that a series of nutritional factors such as protein, calcium, phosphate, or vitamin D can evoke magnesium imbalances, while excess intake of sugar, alcohol, or salt can trigger magnesium losses through enhanced urinary excretion or interference of fiber with magnesium absorption [48].

Although the average sodium intake of vegetarians and omnivores was more than 80% above the DRV, the intake values were comparable in magnitude to Clarys et al. (2014), reporting average intake values of about 2300 mg/d for vegetarians and 3300 mg/d for omnivores [7]. A high sodium intake is associated with hypertension and coronary heart disease. Cohort studies have shown that an elevated salt intake of 5 g per day, corresponding to 2000 mg of sodium, increases the overall risk of cardiovascular disease by 17% and of stroke by 23% [49].

For potassium, iodine, and selenium, both vegetarians and omnivores had on average lower intakes than the respective DRV. A Western diet is associated with lower potassium intakes due to reduced consumption of potassium-rich foods such as fruit and vegetables along with an increased sodium intake [50].

In view of the low concentrations of iodine and selenium in European soils and the fact that bread and dairy products constitute the principal sources of iodine in industrialized countries [51], a mild undersupply of these trace elements was to be expected.

At first glance, vegetarians showed on average a sufficient iron and zinc intake according to the DRV of 15 mg/d, respectively, 8 mg/d. In plant-based diets, however, it has been shown that the intestinal absorption of iron can be reduced by up to 70% and of zinc by 35% compared to meat eaters in part due to increased intakes of phytate from vegetables and whole grains, acting as an absorption inhibitor [52]. Therefore, vegetarians might be at greater risk of developing an iron-deficiency in the long-term compared to omnivores, which could be prevented by iron supplementation. Although 26% of the vegetarians of this study reported having supplemented iron, their intake barely met the intake recommendations, which might favor a deficiency in the long term. A deficit in iron intake has already been shown in a German cross-sectional study investigating the nutrition and health status of students in the city of Schwäbisch Gmünd (*EGS* study) [53].

Focusing on vitamins, vegetarians had significantly lower intakes of retinol as compared to omnivores. Although it is known that an adequate intake of retinol is especially problematic for ovo-lacto-vegetarians [37], the average intake of retinol equivalents in the current study substantially exceeded the DRV of 800 µg/d (1 µg retinol equivalents corresponds to 1 µg retinol and 6 µg beta-carotene [6]). Compared to previous publications, such as the *EPIC-Oxford* study involving over 60,000 subjects from the UK, which reported a median intake of retinol in female vegetarians of 277 ± 180 µg/d, similar to this study, it was about 60% lower than for meat eaters (654 ± 617 µg/d) [37]. In a representative subcohort of the *British Columbia Nutrition Survey (BCNS)*, dietary intake of retinol equivalents was indicated with 1208 ± 175 µg/d in self-reported female vegetarians (*n* = 106) and with 1186 ± 53 µg/d in non-vegetarians (*n* = 1711) [54], which corresponds well with present findings. Particularly with regard to the retinol equivalent beta-carotene, vegetarians of the present study had significantly higher intakes compared to omnivores, most likely related to a higher fruit and vegetable consumption.

In contrast to omnivores, the average intake of vegetarian students investigated in this study did not meet the DRV of cobalamin set at 4 µg/d. Given the high concentration of cobalamin in meat and animal foods (e.g., dairy products) as the main dietary sources of cobalamin [55], this result was not particularly surprising. Despite the existence of several alternate cobalamin sources (e.g., edible algae or cyanobacteria, tea leaves, fortified foods, or supplements) [56], these were of little relevance for this research. A closer look at the information provided in the FFQs of vegetarians in this study revealed dairy products, eggs, and fish to be the main sources of cobalamin in their diet. The low cobalamin intakes in vegetarians are in contrast to a study published in 2011 by Schweter et al. who examined the nutritional behavior of 102 female students in Germany using a validated 3-day estimated dietary record and did not detect inadequate cobalamin intakes from the diet [53]. Only 36% of the vegetarians in the present study exhibited an adequate cobalamin intake according to the German DRV, while 42% of the subjects reached at least 67% of the DRV, and 22% of the subjects reported intakes even below. Compared to omnivores, this study found that vegetarians were 0.3 times less likely to exceed the DRV for cobalamin intake than omnivores. It has been widely reported that vegetarians from around the world suffer from insufficient dietary cobalamin intakes and corresponding deficiencies [57,58,59,60,61], which is consistent with the data presented, even though the analysis of functional biomarkers for cobalamin deficiency was not considered herein. In contrast to the *German National Nutrition Survey* (*NVS II*; 2005–2007), which detected a median dietetic cobalamin intake of 4 µg/d for women living in private households in the age group of 25 to 51 years (*n* = 3666), thus pointing to an adequate vitamin intake [62,63], this investigation showed an insufficient cobalamin supply especially in vegetarian women.

Moreover, the *NVS II* revealed inadequate intakes of folate in women (median: 257 µg/d) and classified the intakes of vitamin D as critical [63]. Both vegetarians and omnivores in the current study were on average only slightly above the DRV for folate of 300 µg/d.

Regarding vitamin D, estimates assume that about 10% of this vitamin is ingested through food, whereas approximately 90% is formed from endogenous synthesis in the human skin due to solar radiation (UV-B) [64]. In view of a DGE reference level of 20 µg/d for vitamin D without considering the endogenous synthesis, the cholecalciferol intake within the investigated student cohort by taking into account additional supplementation, was found to be adequate.

While the *NVS II* provided no evidence of an insufficient dietary intake of other vitamins for the German population on average, a marginal undersupply of pantothenic acid, slightly below the recommended intake of 6 mg/d, was detected in female vegetarians and omnivores in the present study. To date, comprehensive data on the content of pantothenic acid in foods are limited. Primary sources of pantothenic acid are quite diverse foods such as meat, codfish, potatoes, whole grains, egg yolk, and tomato products [65]. The bioavailability of pantothenic acid from food is assumed to range from approximately 40 to 60% [62], which in the longer term could foster pantothenic acid deficiency in both omnivores and vegetarians.

For other B vitamins such as pyridoxine, biotin, folic acid, as well as ascorbic acid and alpha-tocopherol, the dietary intake did not differ significantly between vegetarians and omnivores, while the respective DRV were consistently met.

## 5. Strengths and Limitations

A major strength of this study is that it involved a very homogeneous subpopulation, all of whom shared the same gender, age structure, economic situation, as well as educational level. In addition, the focus was on younger female students pursuing a vegetarian lifestyle, on which data on nutrient intake and nutritional status in Germany are currently scarce. An added benefit is also the usage of the well-established validated graphical FFQ, which was originally developed for assessing the habitual food intake in the *Food4Me* study and has successfully been applied on a pan-European scale. The use of FFQs as valid tools for detecting differences in dietary intake of foods and nutrients between subpopulations is well established and scientifically accepted [66].

Furthermore, the results of this study are very promising in the context of the Quisper platform and the delivery of scientifically validated personalized nutrition (PN) advice [15]. The combined use of an FFQ, such as the eNutri2019, and a PN algorithm that provides tailored nutrition advice to consumers, can potentially have a higher impact in nudging people towards healthier (or possibly more sustainable) dietary choices like, for example, a vegetarian diet or plant-based diet. However, the approach will have to be further tested and analyzed in a real world scenario covering all the different population groups [14].

This survey is limited by the fact that the sample of the study is not representative for the general population in Germany, as only a limited number of respondents with mainly higher educational levels was considered, and a disproportionately high share of study participants were residents in Southern Germany. Although the FFQ was designed to retrospectively track the eating habits of study participants with maximum accuracy, a bias due to under- and over-reporting of subjects cannot be excluded. The large number of food items surveyed, as well as the long recall period of 1 month, might also have negatively affected the discriminatory power of the FFQ applied. Additionally, it is important to note that the present study only addressed the dietary intake of foods and nutrients, without considering interactions between food constituents that may affect bioavailability and without taking into account metabolic differences among study participants.

## 6. Conclusions

The objective of this paper was to evaluate the food intake of female vegetarian students in Germany. The performed research demonstrates that vegetarian students tend to eat healthier diets than omnivorous students, which is reflected by an insignificantly higher HEI-2015 score in the vegetarian group. A higher intake of wholegrain products, as well as an increased intake of fruit and vegetables, for example, can be seen as factors contributing to a better HEI-2015 score. However, participants on a vegetarian diet did not meet the DRV for all nutrients. Especially for nutrients which are mainly found in animal products (e.g., cobalamin), an insufficient intake was observed on average. To efficiently prevent nutrient deficiencies, subsequent studies are needed to further evaluate the nutrient supply of vegetarians in Germany. Therefore, it is necessary to not only apply FFQs, but also to capture essential blood biomarkers that supplement insight into the actual nutrient supply in human subjects. Overall, the eNutri2019 FFQ seems to be a suitable dietary assessment tool as a basis for personalized dietary advices to individuals.

## Figures and Tables

**Table 1:** Study population characteristics.

Variable	Vegetarians (*n* = 31)		Omnivores (*n* = 30)	
	% of Participants	Mean ± SD	% of Participants	Mean ± SD
**Age (years)**		25.0 ± 17.7		20.0 ± 14.1
**BMI (kg/m²)** ^a^		21.9 ± 7.0		22.5 ± 0.8
**BMI classification** ^a^				
Underweight (<18.5 kg/m^2^)	12.9		0	
Normal weight (≥18.5 to ≤24.9 kg/m^2^)	77.4		83.3	
Overweight (≥25.0 kg/m^2^)	9.7		16.7	
**Physical activity levels** ^b^				
Inactive	6.5		10.0	
Moderately inactive	16.1		10.0	
Moderately active	12.9		16.7	
Active	64.5		63.3	
Place of residence				
Northern Germany ^c^	9.6		13.3	
Western Germany ^d^	19.4		6.7	
Eastern Germany ^e^	19.4		20.0	
Southern Germany ^f^	51.6		60.0	
**Household net income**				
Not specified	19.4		20.0	
No income	19.4		23.3	
<10.000 €/year	41.9		26.7	
10.000–24.999 €/year	9.7		10.0	
25.000–39.999 €/year	6.5		13.3	
40.000–54.999 €/year	0.0		3.3	
>55.000 €/year	3.2		3.3	
**Dietary supplementation**				
Omega-3, fish oil or cod liver oil	3.2		6.7	
Vitamin C	9.7		10.0	
Vitamin D	25.8		13.3	
Calcium	0.0		6.7	
Iron	25.8		20.0	

^a^ Body Mass Index (BMI) based on self-reported weight and height of study participants; ^b^ Physical activity levels are based on the *General Practice Physical Activity* *Questionnaire* (*GPPAQ*); ^c^ Northern Germany includes the federal states Lower Saxony, Schleswig-Holstein, Hamburg, Bremen; ^d^ Western Germany: North Rhine-Westphalia, Rhineland-Palatinate, Saarland; ^e^ Eastern Germany: Mecklenburg-Western Pomerania, Saxony-Anhalt, Brandenburg, Berlin, Thuringia, Saxony; ^f^ Southern Germany: Bavaria, Baden-Wuerttemberg, Hesse.

**Table 2:** Energy and nutrient intakes of female German students.

Nutritional Information	Vegetarians (*n* = 31)		Omnivores (*n* = 30)		*p* Value ^a^	Dietary Reference Values (DRV) ^b^
	Mean ± SD	Median (IQR) ^c^	Mean ± SD	Median (IQR) ^c^		per Day
Energy (kcal)	1993.5 ± 714.1	1905.1 (2594-1545)	2070.9 ± 764.2	2089.4 (2767-1535)	0.60	2150
Energy (kJ)	8352.6 ± 2989.4	7975.0 (10863-6482)	8675.1 ± 3201.3	8750.0 (11590-6427)	0.61	No DRV
Protein (%TE) ^d^	15.6 ± 3.1	15.2 (19-13)	16.2 ± 3.3	15.6 (18-15)	0.58	No DRV
Protein (g)	79.0 ± 35.8	72.2 (105-51)	84.3 ± 37.9	80.9 (106-54)	0.57	48
Fat (%TE)	29.3 ± 6.1	29.6 (34-25)	33.4 ± 5.0	32.1 (37-30)	0.01 *	30
Fat (g)	67.1 ± 33.2	64.0 (91-39)	75.7 ± 26.4	73.3 (98-60)	0.16	No DRV
SFA (g) ^e^	24.8 ± 14.7	21.4 (33-15)	31.5 ± 12.1	30.6 (39-23)	0.02 *	No DRV
SFA (%TE)	10.6 ± 3.3	10.6 (12-9)	13.9 ± 3.0	13.5 (16-12)	0.0001 ***	10
C16:0 (g)	12.1 ± 6.9	11.0 (17-7)	14.6 ± 5.4	14.7 (18-11)	0.01 *	No DRV
C18:0 (g)	4.3 ± 2.6	3.6 (6-2)	6.5 ± 2.7	5.8 (9-4)	0.002 **	No DRV
MUFA (g) ^f^	22.6 ± 12.5	19.2 (31-13)	25.2 ± 8.7	24.7 (32-20)	0.15	No DRV
MUFA (%TE)	9.7 ± 2.7	9.8 (12-8)	11.2 ± 2.6	10.8 (12-10)	0.02 *	No DRV
PUFA (g) ^g^	15.3 ± 7.0	14.6 (21-10)	13.8 ± 7.4	13.5 (17-9)	0.32	No DRV
PUFA (%TE)	7.0 ± 2.3	6.0 (9-5)	6.0 ± 1.9	5.4 (7-4)	0.07	No DRV
n6-PUFA (g) ^h^	12.9 ± 6.4	11.9 (17-8)	11.4 ± 5.7	11.3 (15-7)	0.36	No DRV
n6-PUFA (%TE)	5.9 ± 2.2	5.6 (7-4)	5.0 ± 1.5	4.5 (6-4)	0.10	2.5
n3-PUFA (g) ^i^	2.2 ± 1.0	2.0 (2.9-1.6)	2.3 ± 1.5	2.0 (2.5-1.6)	0.88	No DRV
n3-PUFA (%TE)	1.0 ± 0.3	1.0 (1.2-0.8)	0.8 ± 0.1	1.0 (1.1-0.7)	0.26	0.5
LC-n3-PUFA (g) ^j^	0.1 ± 0.2	0.1 (0.2-0)	0.2 ± 0.2	0.2 (0.4-0.1)	0.004 **	No DRV
Cholesterol (mg)	196.8 ± 157.4	135.2 (270-65)	282.7 ± 161.1	238.3 (382-168)	0.013 *	No DRV
Fiber (g) ^k^	34.7 ± 12.6	33.1 (48-27)	26.3 ± 12.4	25.7 (31-17)	0.01 **	30
Starch (g)	138.7 ± 58.3	134.8 (169-106)	125.5 ± 57.6	123.8 (144-81)	0.38	No DRV
Carbohydrates (%TE)	46.8 ± 6.3	47.9 (51-41)	43.9 ± 5.6	45.2 (48-41)	0.05	50
Carbohydrates (g)	243.7 ± 80.9	229.7 (299-203)	245.4 ± 103.4	242.9 (112-168)	0.97	No DRV
Total sugars (%TE)	18.3 ± 5.0	18.3 (22-15)	19.9 ± 6.0	18.8 (24-16)	0.39	No DRV
Glucose (g)	19.4 ± 7.9	18.5 (24-14)	18.4 ± 8.4	17.5 (23-12)	0.56	No DRV
Sucrose (g)	37.8 ± 20.1	28.5 (58-22)	54.8 ± 38.7	50.3 (68-26)	0.046 *	No DRV
Lactose (g)	9.6 ± 12.0	5.5 (13-2)	14.9 ± 13.4	10.3 (19-6)	0.02 *	No DRV
Fructose (g)	24.3 ± 12.7	23.4 (31-13)	21.0 ± 12.0	19.1 (27-12)	0.27	No DRV
Calcium (mg)	1137.9 ± 548.5	1004.6 (1440-727)	1208.6 ± 733.8	1074.3 (1546-715)	0.91	1000
Magnesium (mg)	524.6 ± 188.0	502.9 (652-392)	429.8 ± 221.8	381.0 (564-294)	0.03 *	300
Phosphorus (mg)	1579.1 ± 672.0	1436.6 (2133-1099)	1587.1 ± 726.8	1473.4 (2248-1010)	0.99	700
Sodium (mg)	2771.8 ± 1134.8	2747.2 (3622-1823)	2774.6 ± 1211.1	2627.9 (3260-1862)	0.83	1500
Potassium (mg)	3740.6 ± 1267.6	3665.5 (4592-2882)	3347.4 ± 1528.0	3077.8 (4117-2162)	0.16	4000
Iodine (µg)	130.5 ± 62.1	116.7 (164-92)	117.8 ± 55.3	108.6 (144-75)	0.37	200
Iron (mg)	16.3 ± 5.9	15.0 (22-12)	13.8 ± 6.4	12.8 (17-10)	0.08	15
Zinc (mg)	12.6 ± 5.0	11.4 (17-9)	12.2 ± 4.9	11.5 (16-8)	0.97	8.0
Selenium (µg)	6.8 ± 7.0	4.3 (10-2)	11.7 ± 15.8	7.3 (12-3)	0.17	60
Beta-carotene (µg)	6204.6 ± 3595.0	5224.5 (7308-3653)	4807.0 ± 5147.8	3228.8 (4720-2278)	0.01 **	4800
Retinol (µg) ^l^	330.2 ± 251.0	267.2 (499-134)	614.4 ± 479.0	486.4 (747-292)	0.0021 **	No DRV
Retinol equivalents (µg)	1383.3 ± 676.6	1294.6 (1723-884)	1455.1 ± 1118.8	1155.9 (1682-795)	0.56	800
Thiamine (mg)	1.8 ± 1.0	1.7 (2.1-1.2)	1.8 ± 1.3	1.4 (2.3-1.0)	0.82	1.0
Riboflavin (mg)	2.1 ± 0.9	1.7 (2.7-1.6)	2.3 ± 1.7	2.0 (2.4-1.3)	0.58	1.1
Niacin (mg)	17.0 ± 9.2	14.3 (21-11)	18.0 ± 10.5	15.6 (45-23)	0.72	No DRV
Niacin equivalents (mg)	31.6 ± 13.1	30.7 (38-22)	33.8 ± 15.4	30.6 (45-23)	0.76	12.5
Pantothenic acid (mg)	5.4 ± 2.4	4.7 (7-4)	5.6 ± 2.8	5.1 (6-3)	0.82	6.0
Pyridoxine (mg)	1.9 ± 0.8	1.9 (2.6-1.3)	2.0 ± 1.7	1.8 (2.4-1.5)	0.77	1.4
Biotin (µg)	63.5 ± 29.2	63.5 (84-42)	56.3 ± 27.6	49.7 (69-35)	0.32	30-60
Folic acid (µg)	354.6 ± 129.0	351.9 (461-265)	304.0 ± 150.7	287.9 (342-216)	0.08	300
Cobalamin (µg)	3.5 ± 2.3	3.3 (5-2)	5.6 ± 3.6	4.7 (6-3)	0.01 **	4.0
Ascorbic acid (mg)	152.0 ± 75.7	138.8 (192-106)	136.7 ± 69.9	123.3 (177-86)	0.41	95
Cholecalciferol (µg)	8.9 ± 8.8	5.9 (11-4)	7.8 ± 9.5	4.2 (7-4)	0.30	20
Alpha-Tocopherol (mg)	13.9 ± 5.8	13.2 (20-9)	12.1 ± 5.5	11.3 (16-9)	0.28	12
Alcohol (g)	11.2 ± 17.5	5.4 (11-0.2)	6.7 ± 6.9	4.4 (12-0.8)	0.72	10

^a^*p* value for comparison of Medians: * *p* ≤ 0.05, ** *p* ≤ 0.01, *** *p* ≤ 0.001; Mann–Whitney-U test; ^b^ Intake recommendations according to the DGE [6] (average DRV values for female age groups 19–25 years and 25–51 years, assuming an average activity level for energy intake [PAL 1.6], an average phytate intake for zinc, and neglecting the endogenous synthesis of cholecalciferol); ^c^ IQR, Inter-quartile range, 75–25th percentile; ^d^ %TE: Percent of total energy; ^e^ SFA: Short-chain fatty acids; ^f^ MUFAs: Monounsaturated fatty acids; ^g^ PUFA: Polyunsaturated fatty acids; ^h^ Omega-6 series of PUFA; ^i^ Omega-6 series of PUFA; ^j^ LC: Long-chain; ^k^ AOAC fiber: Includes NSP and non-digestible carbohydrates; ^l^ 1 mg retinol corresponds to 1 mg retinol equivalents.

**Table 3:** Healthy Eating Index-2015 (HEI-2015) of female students.

Components	Maximum Scores	Scoring Vegetarians (*n* = 31)		Scoring Omnivores (*n* = 30)		*p* Value ^d^
		Mean ± SD	Median (IQR) ^c^	Mean ± SD	Median (IQR) ^c^	
***Adequacy*** ^a^						
Total fruits ^1^	5	4.2 ± 1.7	5.0 (5-5)	4.3 ± 1.5	5.0 (5-5)	0.82
Whole fruits ^2^	5	4.1 ± 1.7	5.0 (5-4)	4.0 ± 1.6	5.0 (5-3.4)	0.94
Total vegetables	5	4.8 ± 0.8	5.0 (5-5)	4.5 ± 1.3	5.0 (5-5)	0.36
Greens & Beans ^3^	5	5.0 ± 0.2	5.0 (5-5)	4.6 ± 1.2	5.0 (5-5)	0.06
Whole Grains	10	9.7 ± 1.4	10.0 (10-10)	9.3 ± 2.1	10.0 (10-10)	0.30
Dairy ^4^	10	5.4 ± 4.3	4.8 (10-1.1)	8.0 ± 3.1	10.0 (10-7.1)	0.01 **
Total Protein Foods	5	2.8 ± 0.6	2.7 (3.2-2.3)	2.9 ± 0.6	2.8 (3.1-2.6)	0.63
Seafood & Plant Proteins ^5^	5	4.8 ± 0.7	5.0 (5-5)	3.9 ± 1.4	4.9 (5-2.6)	0.001 **
Fatty Acids ^6^	10	6.7 ± 2.0	6.5 (8-4.9)	5.1 ± 1.5	4.7 (6-4.1)	0.001 **
***Moderation*** ^b^						
Refined Grains	10	9.3 ± 0.9	9.7 (10-8.8)	9.4 ± 0.7	9.6 (10-9.1)	0.71
Sodium	10	6.7 ± 2.8	7.0 (9.1-5)	6.9 ± 2.7	7.3 (9.4-5)	0.92
Added Sugars ^7^	10	9.3 ± 1.1	10.0 (10-8.8)	7.9 ± 2.3	8.4 (10-7.1)	0.003 **
Saturated Fats	10	6.5 ± 3.3	6.7 (9.3-5.2)	3.3 ± 2.7	3.1 (5.6-0.6)	0.0002 ***
***Total Score***	100	79.0 ± 16.0	82.5 (100-66.3)	73.9 ± 17.7	80.7 (100-63.8)	0.83

^a^ Adequacy components: Higher scores reflect higher intakes and are desirable; ^b^ Moderation components: Lower scores reflect lower intakes and are desirable; ^c^ IQR, Inter-quartile range, 75–25th percentile; ^d^ *p* value for comparison of Medians: * *p* ≤ 0.05, ** *p* ≤ 0.01, *** *p* ≤ 0.001; Mann–Whitney-U test; ^1^ Includes all forms of fruit including fruit juice; ^2^ Includes all forms of fruit except fruit juice; ^3^ Includes legumes, beans, and peas; ^4^ Includes all milk products (e.g., fluid milk, yogurt, cheese and fortified soy beverages); ^5^ Includes seafood, nuts, seeds, soy products (no beverages), beans, and peas; ^6^ Ratio of poly- and mono-unsaturated (PUFA and MUFA) to saturated fatty acids (SFA); ^7^ Sugar added to food during preparation, processing or at table.

## Data Availability

Not applicable.
